# Nasal colonization, risk factors, and antibiotic resistance of methicillin-resistant *Staphylococcus aureus* in Gabonese hospitals

**DOI:** 10.3389/fmicb.2026.1818642

**Published:** 2026-06-09

**Authors:** Bely Fredicienne Gondi Nzamba, Barthelemy Ngoubangoye, Desire Otsaghe Ekore, Annicet-Clotaire Dikoumba, Jean Nzue Nguema, Eva Cleone Ntsaga Matamba, Yasmine Okomo Nguema, Serge-Ely Dibakou, Water Matangoye, Dominique Pontier

**Affiliations:** 1 Centre International de Recherches Médicales de Franceville (CIRMF), Franceville, Gabon; 2 Ecole Doctorale Régional en Infectiologie Tropicale (EDR), Franceville, Gabon; 3 LabEx ECOFECT, Eco-Evolutionary Dynamics of Infectious Diseases, University of Lyon, Lyon, France; 4 CNRS, Laboratoire de Biométrie et Biologie Evolutive UMR5558, Université de Lyon 1, Villeurbanne, France; 5 Ecole de Science et Médecine Vétérinaire de Masuku (ESMVM), Université des Sciences et Techniques de Masuku (USTM), Franceville, Gabon; 6 Département de Biologie Médicale, Hôpital d’Instruction des Armées Omar Bongo Ondimba, Libreville, Gabon; 7 Département de Microbiologie, Université des Sciences et Techniques de la Santé, Libreville, Gabon

**Keywords:** MRSA, *Staphylococcus aureus*, nasal carriage, Panton-Valentine leukocidin, antimicrobial resistance, multidrug resistance, risk factors, Gabon

## Abstract

**Background:**

Methicillin-resistant *Staphylococcus aureus* (MRSA) is a leading cause of nosocomial infections and is associated with increased morbidity, mortality, and healthcare costs. Nasal carriage is a key reservoir for transmission and an important target for infection prevention. Data on MRSA prevalence, risk factors, and Panton–Valentine leucocidin (PVL) associated virulence in Central Africa, particularly in Gabon, remain limited.

**Methods:**

A cross-sectional study was conducted among 636 patients in seven Gabonese hospitals. Nasal swabs were collected for culture and identification of *S. aureus*. Methicillin resistance (*mecA*) and the *LukS/F-PV* gene were detected using polymerase chain reaction (PCR). Antibiotic susceptibility was assessed using the disk diffusion method. Demographic and clinical risk factors were analyzed using univariate and multivariate statistical analyses.

**Results:**

*S. aureus* carriage was detected in 38.2% (243/636) of patients, of whom 47.7% (116/243) had MRSA strains. The PVL gene was detected in 25.5% (62/243) of isolates and was more frequently found in MRSA strains (31.9%) than methicillin-susceptible *S. aureus* (MSSA) (19.7%, *p* = 0.042). No significant associations with age or sex were observed for MRSA carriage. A multivariate analysis identified admission to specific hospitals and the dry season as independent risk factors for MRSA carriage. Among MRSA isolates, high resistance was observed to tetracycline (58.6%) and rifampicin (37.9%), with 37.1% being multidrug-resistant (MDR). Resistance to linezolid (25.9%) was also noted.

**Conclusion:**

MRSA and PVL-positive strains are highly prevalent in Gabonese hospitals and are associated with specific institutional and environmental factors rather than patient-related characteristics. The high proportion of MDR strains and the emergence of linezolid resistance highlight the need to strengthen infection control measures, maintain continuous surveillance, and ensure rational antibiotic use to limit the spread of these virulent clones.

## Introduction

*Staphylococcus aureus* is a Gram-positive bacterium that commonly colonizes human mucous membranes but may cause a wide spectrum of infections, ranging from skin and soft tissue infections to life-threatening conditions such as osteomyelitis, bacteremia, endocarditis, and meningitis (Lowy, 1998). The emergence of methicillin-resistant *S. aureus* (MRSA) is currently a leading cause of both nosocomial and community-acquired infections worldwide. MRSA infections are associated with increased morbidity, mortality, and substantial healthcare costs, posing a significant burden on healthcare systems globally (Turner et al., 2019; European Centre for Disease Prevention and Control, 2017). Furthermore, the ability of MRSA strains to colonize patients and healthcare workers, particularly in the anterior nasal cavity, constitutes a persistent reservoir that facilitates transmission within healthcare facilities (Wertheim et al., 2005).

The global spread of MRSA exhibits genetic diversity and the heterogeneous distribution of epidemic clones. While the prevalence and molecular epidemiology of MRSA are well-documented in North America, Europe, and Asia, data from sub-Saharan Africa remain sparse and fragmented (Kpeli et al., 2017; Schaumburg et al., 2011). This lack of comprehensive data hampers the development of effective control strategies and antibiotic policies tailored to local contexts. In Gabon, as in other Central African countries, the inappropriate use of antibiotics and infrastructural challenges within healthcare systems may contribute to the emergence and dissemination of multidrug-resistant bacteria (Tadesse et al., 2017). Additionally, cross-border population movements, particularly in Gabonese border towns, may facilitate the introduction and exchange of bacterial clones, further complicating infection control efforts (Deplano et al., 2000).

The Panton–Valentine leukocidin (PVL) gene, which encodes a bicomponent cytotoxin produced by certain strains of *S. aureus*, is strongly associated with necrotizing skin infections, soft tissue infections, and severe necrotizing pneumonia. While historically linked to community-acquired *S. aureus* strains (CA-MRSA), recent reports have indicated an increasing prevalence of PVL-positive isolates among hospital-acquired MRSA (Ayepola et al., 2018). The prevalence of PVL-positive strains varies widely across geographic regions and populations (Gillet et al., 2002); however, the clinical significance of these strains in Gabonese hospitals remains poorly characterized. This knowledge gap limits the understanding of the virulence potential of circulating MRSA strains in the region.

This study aimed to (i) determine the prevalence of MRSA nasal colonization among patients admitted to Gabonese hospitals in order to provide essential baseline data for local surveillance programs; (ii) identify risk factors associated with colonization to inform targeted prevention strategies; (iii) assess the presence of the PVL gene in MRSA isolates to evaluate the potential for severe infections and guide clinical management; and (iv) characterize the antibiotic resistance profile of MRSA isolates to support the development of evidence-based antibiotic policies. The findings of this study may contribute critical insights to inform the design of surveillance programs and infection control measures tailored to the Gabonese hospital context.

## Materials and methods

### Ethical considerations

This study is part of the ECOPAR (Parasite Ecology) project, approved by the National Ethics and Research Committee (CNER) in Gabon. The project, which complies with the Declaration of Helsinki, was validated (protocol PROT/0020/2013/SG/CNER), and authorization was also obtained from the management of each Regional, University, and Military Hospital Centre. All participants were informed about the importance of the study for the surveillance of nosocomial strains and the procedures for sample collection. Informed verbal consent was obtained from each participant, and parental consent was obtained for children.

### Sampling sites

This study was conducted in seven major hospitals in Gabon (population 1,811,079, of whom more than 93% live in urban areas), located across five of the country’s nine provinces and representing both urban and regional healthcare facilities. The hospitals included the Omar Bongo Ondimba Armed Forces Teaching Hospital (HIAOBO), the Akanda Armed Forces Teaching Hospital (HIAA), and the University Hospital Centre (CHUL) in Libreville (the capital, which accounts for 49.5% of the population); the Georges Rawiri Regional Hospital Centre (CHRGR) in Lambaréné; the Benjamin Ngoubou Regional Hospital Centre (CHRBN) in Tchibanga; the Amissa Bongo University Hospital Centre (CHRAB); and the Oyem Regional Hospital Centre (CHRO). These hospitals have a combined total bed capacity of 1,491. The geographic distribution of these hospitals allowed this study to analyze social determinants, including the province of residence, urban vs. rural setting, hospital and department of admission, and occupation, which are relevant for assessing MRSA and PVL colonization risk.

### Patient recruitment and sampling

Samples were collected over 1 month at each hospital, between February and July 2024. Demographic and clinical information, including age, sex, occupation, region, hospital, department, season, reason for hospitalization, diagnosis or symptoms, and antibiotic use in the 6 months prior to admission, was collected using a structured questionnaire administered to each patient. The exclusion criteria included children under 5 years of age, immunocompromised patients, and intubated patients. Nasal swabs were obtained within 24 h of admission from 100 patients at each hospital (D0 < 24 h). Nasal swabs were collected from the anterior nares using sterile cotton-tipped swabs, inserted approximately 2 cm into each nostril, and rotated five times by trained personnel following aseptic procedures. Samples were homogenized, incubated in brain–heart infusion (BHI) broth at 37 °C for 48 h, and then stored at −20 °C after adding 50% glycerol.

### Isolation and identification of *Staphylococcus aureus*

The bacterial suspensions were inoculated in mannitol agar (BioMérieux, France). Colonies exhibiting the typical characteristics of *S. aureus* (yellow colonies that ferment mannitol) were identified and then subcultured in trypticase soy agar (BioMérieux, France) for confirmation and purification. Mannitol salt agar cultures were incubated at 37 °C for 48 h, while those on trypticase soy agar were incubated for 18–24 h.

### Extraction and amplification of *Staphylococcus aureus* genes *mec*A and *luks/F-PV*

DNA characteristic colonies of *S. aureus* were extracted using the heat shock method (Hesari et al., 2018). The polymerase chain reaction (PCR) was performed to detect the *16S rRNA*, *nuc*, *mecA,* and *lukS/F-PV* genes in *S. aureus*. Amplification of these genes was performed in a total volume of 25 μL, consisting of 12.5 μL AmpliTaq Gold Master Mix (Applied Biosystems, Thermo Fisher Scientific, Waltham, MA, USA), 6 μL ultrapure water, and 0.75 μL of each primer (20 μM). Detection of the *lukS/F-PV* gene was performed on all strains identified as *S. aureus*, using the same amplification conditions as for the *nuc* gene. The specific primers used for amplification and detection of the different genes are listed in Table 1.

**Table 1:** Primer sequences.

Target gene	Primer sequence (5′ → 3′)	T^°^C	Product size (bp)	References
16S rRNA	AAAGCGATTGATGGTGATACGGTT	56	600	Gondi Nzamba et al. (2025)
	TGCTTTGTTTCAGGTGTATCAACCA			
*mec*A	GTGAAGATATACCAAGTGATT	61	533	Gondi Nzamba et al. (2025)
	ATGCGCTATAGATTGAAAGGAT			
*LukS/F-PV*	ATCATTAGGTAAAATGTCTGGACATGATCCA	56	433	Velasco et al. (2014)
	GCATCAAGTGTATTGGATAGCAAAAGC			
*nuc*	GCGATTGATGGTGATACGGTT	58	270	Ngoubangoye et al. (2023)
	AGCCAAGCCTTGACGAACTAAAGC			

### Antimicrobial susceptibility testing

Antimicrobial susceptibility testing of all isolated MRSA strains was performed following the recommendations of the European Committee on Antimicrobial Susceptibility Testing (EUCAST, 2025) using the disk diffusion method. The antibiotics tested included chloramphenicol (CHL, 30 μg), ciprofloxacin (CIP, 5 μg), clindamycin (CLI, 2 μg), linezolid (LNZ, 10 μg), rifampicin (RIF, 5 μg), tetracycline (TET, 30 μg), and trimethoprim-sulfamethoxazole (SXT, 1.25/23.75 μg). Multidrug-resistant (MDR) strains were defined as those resistant to at least three different classes of antibiotics (Magiorakos et al., 2012).

### Statistical analyses

In this study, categorical data were expressed as frequencies and percentages, while normally distributed data were expressed as means ± standard deviation. Comparisons between MRSA and methicillin-sensitive *S. aureus* (MSSA) carriers were performed using the χ^2^ test for categorical variables and the *t*-test or Wilcoxon test for continuous variables, as appropriate. No adjustment for multiple comparisons was applied in the univariate analyses, consistent with exploratory analysis, and variables with a *p*-value < 0.20 were included in the multivariate logistic regression model. Unconditional logistic regression models were used to assess risk factors for MRSA and PVL colonization among *S. aureus* carriers. Odds ratios (OR) and 95% confidence intervals (95% CI) were calculated for each factor.

## Results

### Demographic characteristics and prevalence rate

A total of 636 patients were included in this study, of whom 38.2% (*n* = 234/636) were colonized with *S. aureus*. The overall prevalence of MRSA strains in the population was 18.2% (*n* = 116/636). Among the *S. aureus* isolates, 47.7% (*n* = 116/243) were MRSA and 25.5% (*n* = 62/243) were PVL-positive. The sex distribution (47.3% male and 52.7% female) showed no significant difference for MRSA (*p* = 0.428) or PVL-positive strains (*p* = 0.314). The mean age of patients was 31.8 years (±19.1), with no significant variation between the *S. aureus* and PVL-positive groups (*p* = 0.749 and *p* = 0.575). Age group distribution (children < 18 years: 26.9%; adults 18–65 years: 65.3%; and elderly ≥65 years: 7.9%) was also homogeneous between groups (*p* = 0.999 for MRSA and *p* = 0.809 for PVL-positive). Overall, no significant association was observed between the demographic characteristics studied and carriage of MRSA, MSSA, or PVL-positive strains (Table 2).

**Table 2:** Distribution of MRSA and MSSA strains according to demographic characteristics.

Variable	Patient (*n* = 636)	*S. aureus* (*n* = 243)	MRSA (*n* = 116)	MSSA (*n* = 127)	*p*-value	PVL+ (*n* = 62)	*p*-value
Sex					0.428		0.314
Male	301 (47.3%)	114 (46.9%)	58 (50.0%)	56 (44.1%)		33 (53.2%)	
Female	335 (52.7%)	129 (53.1%)	58 (50.0%)	71 (55.9%)		29 (46.8%)	
Age (years)	31.8 (±19.1)	31.8 (±19.6)	32.2 (±19.4)	31.4 (±19.9)	0.749	30.6 (±19.7)	0.575
Age group					0.999		0.809
Children (<18 years)	171 (26.9%)	71 (29.2%)	34 (29.3%)	37 (29.1%)		17 (27.4%)	
Adults (18–65 years)	415 (65.3%)	153 (63.0%)	73 (62.9%)	80 (63.0%)		41 (66.1%)	
Elderly (≥65 years)	50 (7.90%)	19 (7.80%)	9.0 (7.80%)	10 (7.9%)		4.0 (6.50%)	

### Detection of PVL in MRSA and associated factors

A PVL carriage rate of 25.5% (*n* = 62/243) was observed among *S. aureus* isolates, corresponding to 9.6% (*n* = 62/636) of the overall study population. The PVL gene was detected more frequently in MRSA isolates (31.9%, *n* = 37/116) than in MSSA isolates (19.7%, *n* = 25/127), indicating a statistically significant difference (*p* = 0.042).

Overall, the pvl gene was more frequently detected among MRSA (36.4%, *n* = 8/22) than among MSSA (22.5%, *n* = 7/31) isolates. The highest prevalence of the pvl gene was observed among HCWs in the pediatrics department (11.4%, *n* = 7/61), followed by critical care (8.5%, *n* = 5/59), surgery (3.2%, *n* = 1/31) and the lowest prevalence was observed in medicine (2.5%, *n* = 2/79).

No significant differences in clinical characteristics were observed between PVL-positive and PVL-negative patients (all *p* > 0.050). In contrast, PVL carriage was significantly associated with geographical region (*p* = 0.021), recruiting hospital (*p* < 0.001), and season (*p* < 0.001). These findings suggest that PVL carriage is influenced more by specific environmental and institutional factors than by individual patient characteristics (Table 3).

**Table 3:** Univariate analysis of factors associated with PVL carriage.

Variable	PVL-Negative *N* = 79	PVL-Positive *N* = 37	*p*-value
Area			**0.021**
Urban	75 (94.9%)	29 (78.4%)	
Semi-rural	3 (3.8%)	7 (18.9%)	
Rural	1 (1.3%)	1 (2.7%)	
Ward			0.400
Emergency room	25 (31.6%)	17 (45.9%)	
Maternity gynecology	12 (15.2%)	5 (13.5%)	
Medicine	14 (17.7%)	4 (10.8%)	
Pediatrics	11 (13.9%)	7 (18.9%)	
Surgery	17 (21.5%)	4 (10.8%)	
Hospital			**<0.001**
CHRBN	12 (15.2%)	7 (18.9%)	
CHRGR	5 (6.3%)	3 (8.1%)	
CHRO	7 (8.9%)	18 (48.6%)	
CHUAB	5 (6.3%)	7 (18.9%)	
CHUL	17 (21.5%)	0 (0.0%)	
HIA	20 (25.3%)	2 (5.4%)	
HIAA	13 (16.5%)	0 (0.0%)	
Professional_activity			0.300
Retired	4 (5.1%)	5 (13.5%)	
Student	32 (40.5%)	17 (45.9%)	
Unemployed	17 (21.5%)	5 (13.5%)	
Worker	26 (32.9%)	10 (27.0%)	
Season			**<0.001**
Rainy season	60 (75.9%)	12 (32.4%)	
Dry season	19 (24.1%)	25 (67.6%)	
Reason for hospitalization			0.300
Digestive symptom	4 (5.1%)	7 (18.9%)	
General symptom	14 (17.7%)	4 (10.8%)	
Infectious disease	20 (25.3%)	9 (24.3%)	
Local symptom	3 (3.8%)	1 (2.7%)	
Medical condition	13 (16.5%)	3 (8.1%)	
Neurological symptom	2 (2.5%)	1 (2.7%)	
Obstetrics and gynecology	4 (5.1%)	3 (8.1%)	
Respiratory symptom	1 (1.3%)	0 (0.0%)	
Surgical condition	10 (12.7%)	2 (5.4%)	
Trauma and interventions	8 (10.1%)	7 (18.9%)	
Diagnosis			0.800
Established diagnosis	55 (69.6%)	24 (64.9%)	
Presenting symptom	24 (30.4%)	13 (35.1%)	
Antibiotics_six month			>0.900
No	57 (73.1%)	26 (72.2%)	
Yes	21 (26.9%)	10 (27.8%)	

Corresponding acronyms: HIAOBO, the Omar Bongo Ondimba Armed Forces Teaching Hospital; HIAA, the Akanda Armed Forces Teaching Hospital; CHUL, the University Hospital Centre; CHRGR, the Georges Rawiri Regional Hospital Centre; CHRBN, the Benjamin Ngoubou Regional Hospital Centre; CHRAB, the Amissa Bongo University Hospital Centre; CHRO, the Oyem Regional Hospital Centre. Significant associations are indicated with *p*-values in bold.

Overall, the PVL gene was more frequently detected among MRSA (36.4%, *n* = 8/22) in comparison to MSSA (22.5%, *n* = 7/31) isolates. The highest prevalence of the PVL gene was observed from HCWs in the department of pediatrics (11.4%, *n* = 7/61), followed by critical care (8.5%, *n* = 5/59), surgery (3.2%, *n* = 1/31) and the lowest prevalence in medicine (2.5%, *n* = 2/79).

### Risk factors for MRSA carriage

A univariate analysis of the 243 isolates of *S. aureus* (127 MSSA and 116 MRSA) according to demographic and clinical characteristics revealed significant differences with respect to the origin hospital (*p* = 0.002) and the collection season (*p* = 0.012). MRSA was proportionally more frequent in CHRO (21.6% MRSA vs. 10.2% MSSA) and HIA hospitals (19.0% MRSA vs. 8.7% MSSA) and also during the dry season (37.9% MRSA vs. 22.8% MSSA). No significant differences were observed for hospital department, geographical region, reason for hospitalization, type of diagnosis, occupational activity, or antibiotic use in the previous 6 months (*p* > 0.050; Table 4).

**Table 4:** A univariate analysis of factors associated with nasal colonization by MRSA among *S. aureus* carriers.

Variable	MSSA (*n* = 127)	MRSA (*n* = 116)	*p*-value
Hospital			**0.002**
HIAA	30 (23.6%)	13 (11.2%)	
CHUL	25 (19.7%)	17 (14.7%)	
CHRO	13 (10.2%)	25 (21.6%)	
CHUAB	21 (16.5%)	12 (10.3%)	
HIA	11 (8.7%)	22 (19.0%)	
CHRBN	13 (10.2%)	19 (16.4%)	
CHRGR	14 (11.0%)	8 (6.9%)	
Ward			>0.900
Emergency room	41 (32.3%)	42 (36.2%)	
Maternity gynecology	18 (14.2%)	17 (14.7%)	
Medicine	22 (17.3%)	18 (15.5%)	
Pediatrics	21 (16.5%)	18 (15.5%)	
Surgery	25 (19.7%)	21 (18.1%)	
Season			**0.012**
Rainy season	98 (77.2%)	72 (62.1%)	
Dry season	29 (22.8%)	44 (37.9%)	
Area			0.600
Urban	118 (92.9%)	104 (89.7%)	
Semi-rural	8 (6.3%)	10 (8.6%)	
Rural	1 (0.8%)	2 (1.7%)	
Reason for hospitalization			0.200
Infectious disease	46 (36.2%)	29 (25.0%)	
General symptom	16 (12.6%)	18 (15.5%)	
Medical condition	12 (9.4%)	16 (13.8%)	
Surgical condition	15 (11.8%)	12 (10.3%)	
Trauma and interventions	8 (6.3%)	15 (12.9%)	
Digestive symptom	9 (7.1%)	11 (9.5%)	
Obstetrics and gynecology	11 (8.7%)	7 (6.0%)	
Local symptom	7 (5.5%)	4 (3.4%)	
Neurological symptom	0 (0.0%)	3 (2.6%)	
Respiratory symptom	2 (1.6%)	1 (0.9%)	
Various symptom	1 (0.8%)	0 (0.0%)	
Diagnosis/symptoms			0.500
Established diagnosis	92 (72.4%)	79 (68.1%)	
Presenting symptom	35 (27.6%)	37 (31.9%)	
Professional activity			>0.900
Student	54 (42.5%)	49 (42.2%)	
Worker	37 (29.1%)	36 (31.0%)	
Unemployed	27 (21.3%)	22 (19.0%)	
Retired	9 (7.1%)	9 (7.8%)	
Jobseeker	0 (0.0%)	0 (0.0%)	
Antibiotics in the last 6 months	24 (18.9%)	31 (26.7%)	0.150

Corresponding acronyms: HIAOBO, the Omar Bongo Ondimba Armed Forces Teaching Hospital; HIAA, the Akanda Armed Forces Teaching Hospital; CHUL, the University Hospital Centre; CHRGR, the Georges Rawiri Regional Hospital Centre; CHRBN, the Benjamin Ngoubou Regional Hospital Centre; CHRAB, the Amissa Bongo University Hospital Centre; CHRO, the Oyem Regional Hospital Centre. Significant associations are indicated with *p*-values in bold.

A logistic regression analysis identified independent risk factors significantly associated with MRSA carriage, using CHUAB hospital as the reference hospital. Admission to HIA (OR = 10.95; 95% CI: 3.25–41.65; *p* < 0.001), CHRBN (OR = 6.88; 95% CI: 2.07–25.61; *p* = 0.002), and CHUL (OR = 3.66; 95% CI: 1.15–13.01; *p* = 0.034) was associated with an increased risk of MRSA carriage. Additionally, the dry season emerged as a major risk factor (OR = 6.59; 95% CI: 2.38–20.19; *p* < 0.001). Previous antibiotic use within the 6 months prior to admission did not reach statistical significance, although a trend toward increased risk was observed (OR = 1.95; 95% CI: 0.99–3.91; *p* = 0.056; Table 5).

**Table 5:** A multivariate logistic regression analysis of factors associated with MRSA nasal colonization.

Variable	Odds ratio	95% CI lower	95% CI upper	*p*-value
Hospital				
CHUAB	–	–	–	
CHRBN	6.88	2.07	25.61	**0.002**
HIAA	0.91	0.32	2.65	0.859
CHRO	1.62	0.54	4.88	0.385
CHUL	3.66	1.15	13.01	**0.034**
HIA	10.95	3.25	41.65	**<0.001**
CHRGR	2.79	0.73	11.23	0.136
Season				
Rainy	–	–	–	
Dry	6.59	2.38	20.19	**<0.001**
Antibiotic use				
No	–	–	–	
Yes	1.95	0.99	3.91	0.056

CI, 95% confidence interval; HIA, Omar Bongo Ondimba Armed Forces Teaching Hospital; CHRBN, Benjamin Ngoubou Regional Hospital Centre; CHUL, University Hospital Centre; CHUAB, Reference hospital for the analysis. Bold values indicate *p* < 0.05.

### Antibiotic susceptibility and multi-drug resistance

**Figure 1:**
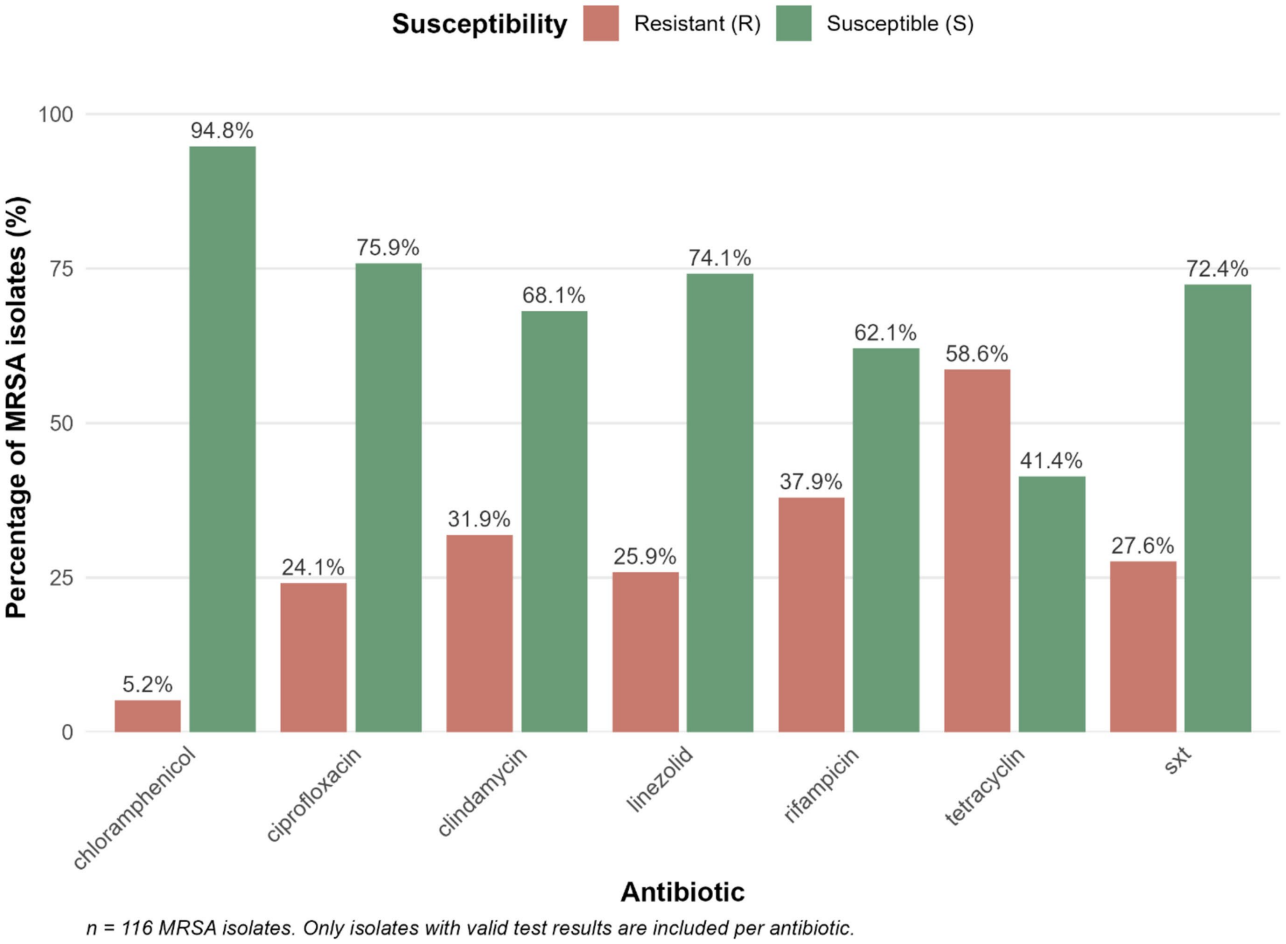
Antibiotic resistance profile of MRSA isolates. SXT: Trimethoprim-sulfamethoxazole. Among the 116 MRSA isolates analyzed, 43 (37.1%) were MDR, exhibiting resistance to at least three antibiotic classes. Resistance limited to one or two classes (non-MDR phenotype) was identified in 51 isolates (44.0%), while 22 isolates (19.0%) were fully sensitive to all tested antibiotic classes. Overall, 81.0% of isolates showed resistance to at least one class of antibiotics, and a substantial proportion displayed an extended resistance phenotype (MDR).

Analysis of overall antibiotic resistance revealed particularly high resistance rates to tetracycline (58.6%, *n* = 68/116). Moderate resistance rates were observed for rifampicin (37.9%, *n* = 44/116), clindamycin (31.9%, *n* = 37/116), trimethoprim–sulfamethoxazole (27.6%, *n* = 32/116), linezolid (25.9%, *n* = 30/116), and ciprofloxacin (24.1%, *n* = 28/116). In contrast, chloramphenicol had a low resistance rate (5.2%, *n* = 6/116; Figure 1).

## Discussion

This study provides the first comprehensive assessment of MRSA nasal colonization in Gabonese hospitals, covering prevalence, associated risk factors, PVL gene carriage, and antibiotic resistance profiles.

### Prevalence and regional comparisons

This is the first large-scale study characterizing MRSA nasal colonization in Gabonese hospitals. The overall colonization rate was 38.2%, which was higher than the rates reported in Japan (22.2%) and Ethiopia (13.6%) but lower than that observed in Nigeria (61.8%) (Kawayanagi et al., 2025; Gebremeskel et al., 2022; Adeiza et al., 2020). Among the 243 *S. aureus* isolates, 116 (47.7%) were MRSA, a prevalence similar to that reported in Nigeria (46.9%), but considerably higher than those observed in other regions of Ethiopia (4.8%; among medical students) and in Brazil (8.8%; among patients with chronic renal failure undergoing hemodialysis) (Adeiza et al., 2020; Shume et al., 2024; Bezerra et al., 2025). These differences likely reflect variations in study populations, sample types, sociodemographic characteristics, clinical histories, antibiotic use practices, screening methods, microbiological analyses, and study periods.

### MRSA–PVL gene association: clinical and epidemiological implications

A significant association between MRSA and PVL carriage was observed (*p* = 0.042), consistent with reports of an increasing prevalence of PVL-positive MRSA isolates in hospital settings, although this prevalence varies considerably across geographical regions and populations (Eckhardt et al., 2003; Linde et al., 2005; Bhatta et al., 2016). This result contrasts with earlier observations in which the PVL gene was primarily associated with CA-MRSA; however, it aligns with more recent evidence of its global emergence among hospital-acquired MRSA strains (Tattevin, 2011).

This co-occurrence of MRSA and PVL may enhance both virulence and transmissibility, highlighting the importance of monitoring PVL-positive MRSA in healthcare environments (Maltezou and Giamarellou, 2006).

No significant association was found between MRSA colonization and age, sex, or other sociodemographic factors. This contrasts with findings from studies conducted in Ecuador, where male sex and older age were identified as risk factors (Baroja et al., 2021).

### Risk factors

The univariate analysis showed that the hospital of admission and season were significantly associated with MRSA and PVL carriage, while geographical region was associated with PVL carriage (*p* < 0.005). A multivariate logistic regression confirmed that admission to CHRBN, CHUL, or HIA, along with the dry season, was an independent risk factor for MRSA colonization. This pattern may be explained by the fact that HIA and CHUL receive a large number of patients referred from other hospitals across the country, including from the five remaining facilities, which may facilitate the transmission of nosocomial bacteria (Maltezou and Giamarellou, 2006).

Recent use of antibiotics was not statistically significantly associated with MRSA colonization in the multivariate model (*p* = 0.056), although a trend toward increased risk was observed, consistent with findings from Nigeria and Spain (Adeiza et al., 2020; Asensio et al., 1996). This could be explained by the high overall prevalence of MRSA, which may mask the specific effect of prior antibiotic exposure, along with limitations related to self-reported data.

### Dry season as a major risk factor

The identification of the dry season as a major risk factor for MRSA nasal colonization (OR = 6.59) is a novel and intriguing finding. While seasonal variations have previously been associated with community-acquired skin infections (Masson, 2026), these results suggest that environmental factors may also influence nasal colonization in Gabon, warranting further investigation. The mechanisms underlying this association remain uncertain and may involve environmental, behavioral, and biological factors.

First, environmental conditions during the dry season (June–September in Gabon) may facilitate MRSA survival and transmission. Lower humidity levels could enhance the persistence of *S. aureus* on surfaces and in dust, as desiccation tolerance varies among bacterial strains, with some MRSA clones exhibiting increased survival in dry environments (Baede et al., 2022). Additionally, the dry season is characterized by increased airborne dust and particulate matter, which may facilitate bacterial dissemination within healthcare facilities, particularly in settings with limited air filtration or ventilation systems (Gupta et al., 2024).

Second, behavioral and healthcare-related factors may also contribute to this association. The dry season may coincide with increased agricultural activity and rural-to-urban migration in Gabon, potentially leading to higher hospital admission rates due to injuries or infections, as reported in other sub-Saharan African settings (Harikripal et al., 2026). Resulting overcrowding in healthcare facilities may amplify nosocomial transmission, particularly in resource-limited contexts where infection control practices are suboptimal (Bankar et al., 2024).

Furthermore, water scarcity during the dry season may compromise hand hygiene practices among healthcare workers and patients, further facilitating MRSA spread.

Third, host-related biological mechanisms may also play a role. Dry environmental conditions may induce mucosal drying and microtrauma in the nasal epithelium, thereby facilitating MRSA colonization. Furthermore, these conditions are supported by evidence showing that epithelial barrier disruption enhances *S. aureus* adhesion and invasion (Pietrocola et al., 2017). Moreover, seasonal variations in immune function, such as reduced mucosal immunity during periods of environmental stress, may increase susceptibility to colonization (MacGillivray and Kollmann, 2014).

Although this study was not designed to elucidate causal pathways, these hypotheses provide a framework for future research. Longitudinal studies integrating environmental monitoring, behavioral data, and molecular characterization of MRSA isolates are needed to understand these dynamics and inform targeted interventions. Such investigations are particularly important in Gabon, where pronounced seasonal variations and constrained healthcare resources may exacerbate transmission risks.

### Antibiotic resistance

Antibiotic susceptibility testing revealed high levels of resistance to tetracycline (58.6%) and rifampicin (37.9%), with moderate resistance to clindamycin, trimethoprim-sulfamethoxazole, linezolid (25.9%), and ciprofloxacin. Notably, the observed rate of resistance to linezolid—a last-resort antibiotic—remains unexpectedly high and should be interpreted with caution. As this study relied on the disk diffusion method without confirmatory minimum inhibitory concentration (MIC) determination or molecular characterization, overestimation cannot be excluded. Although sporadic linezolid resistance has been previously reported, often linked to chromosomal mechanisms (Bourgeois-Nicolaos et al., 2012), further investigations are needed to validate this finding.

These data reflect colonizing strains rather than clinical infections, which may influence resistance patterns. The variability observed across antibiotics suggests that, while some treatments may remain effective against certain strains, others may be increasingly compromised by resistance (Shume et al., 2024). Interestingly, no significant association was detected between recent antibiotic use and MRSA colonization. This lack of association may reflect the dominant role of environmental and institutional factors, such as hospital and seasonal factors, over individual antibiotic exposure in shaping colonization patterns, and limitations inherent to self-reported data.

The high proportion of MDR MRSA strains (37.1%) is particularly concerning and may lead to significant therapeutic challenges. This pattern likely reflects multiple contributing factors, including empirical antibiotic use without prior susceptibility testing, overcrowding in healthcare facilities, and the transmission of resistance determinants. Similar trends have been reported in other African populations, such as Ghana (Kpeli et al., 2017), suggesting that local practices may favor the emergence and dissemination of resistant clones. Additionally, ongoing genetic evolution, horizontal gene transfer, and unregulated antibiotic use may further accelerate the spread of MDR MRSA strains.

## Limitations and future directions

This study has several limitations. The cross-sectional design precludes establishing causal relationships between the identified factors and MRSA or PVL carriage, and the 6-month data collection period may not fully capture seasonal variations or long-term epidemiological trends. Self-reported antibiotic use may be subject to recall bias, potentially affecting associations with MRSA colonization. The absence of advanced molecular characterization (such as whole-genome sequencing or MLST typing) limits the analysis of clonal diversity, resistance, and virulence mechanisms. Additionally, sampling was restricted to nasal swabs, omitting other potential colonization sites (such as skin and oropharynx), and data on hospital hygiene practices and infection control protocols were unavailable, limiting the interpretation of inter-hospital differences in MRSA prevalence. Notably, linezolid resistance should be interpreted cautiously, as only disk diffusion was performed. Future studies should incorporate longitudinal designs, include molecular surveillance, broaden sampling sites, and evaluate infection control practices to validate and extend these findings.

## Conclusion

This first large-scale study in Gabon reveals a concerning situation, with a high prevalence of MRSA strains (47.7% of *S. aureus* carriers) and frequent association with the PVL virulence gene (25.5%). Environmental and institutional factors–particularly the hospital of admission and the dry season–appear to play a more important role than individual characteristics in driving colonization. Resistance patterns are of particular concern, with high rates of tetracycline and rifampicin resistance, emerging linezolid resistance, and a substantial proportion of MDR strains.

Overall, these results highlight the urgent need to strengthen infection control programs, implement continuous microbiological surveillance, promote rational antibiotic use, and integrate molecular epidemiology approaches to monitor, prevent, and limit the spread of resistant and virulent clones in Gabonese hospitals.

## Data Availability

The original contributions presented in the study are included in the article/supplementary material, further inquiries can be directed to the corresponding author/s.

## References

[ref1] Adeiza S. S. Onaolapo J. A. Olayinka B. O. (2020). Prevalence, risk-factors, and antimicrobial susceptibility profile of methicillin-resistant *Staphylococcus aureus* (MRSA) obtained from nares of patients and staff of Sokoto state-owned hospitals in Nigeria. GMS Hyg. Infect. Control. 15:Doc25. doi: 10.3205/dgkh000360, 33214990 PMC7656983

[ref2] Asensio A. Guerrero A. Quereda C. Lizán M. Martinez-Ferrer M. (1996). Colonization and infection with methicillin-resistant *Staphylococcus aureus*: associated factors and eradication. Infect. Control Hosp. Epidemiol. 17, 20–28. doi: 10.1086/647184, 8789683

[ref3] Ayepola O. O. Olasupo N. A. Egwari L. O. Schaumburg F. (2018). Characterization of Panton–Valentine leukocidin-positive *Staphylococcus aureus* from skin and soft tissue infections and wounds in Nigeria: a cross-sectional study. F1000Res. 7:1155. doi: 10.12688/f1000research.15484.1, 30345027 PMC6171726

[ref4] Baede V. O. Tavakol M. Vos M. C. Knight G. M. van Wamel W. J. B. MACOTRA study group (2022). Dehydration tolerance in epidemic versus nonepidemic MRSA demonstrated by isothermal microcalorimetry. Microbiol. Spectrum 10:e0061522. doi: 10.1128/spectrum.00615-22, 35972129 PMC9602581

[ref5] Bankar N. Shelke Y. Bandre G. Kohle M. (2024). “MRSA in hospital setting,” in One Health Approach - Advancing Global Health Security with the Sustainable Development Goals In: Saxena KS, editor. Sustainable Development. (IntechOpen). doi: 10.5772/intechopen.106311

[ref6] Baroja I. Guerra S. Coral-Almeida M. Ruíz A. Galarza J. M. de Waard J. H. . (2021). Methicillin-resistant *Staphylococcus aureus* nasal colonization among health care workers of a tertiary hospital in ecuador and associated risk factors. Infect. Drug Resist. 14, 3433–3440. doi: 10.2147/IDR.S326148, 34471363 PMC8403571

[ref7] Bezerra D. T. Mesquita-Ferrari R. A. Fernandes K. P. S. Bussadori S. K. Motta L. J. Ando-Suguimoto E. S. . (2025). Prevalence of nasal *Staphylococcus aureus* carriage in patients undergoing Hemodialysis and assessment of risk factors: a cross-sectional study of outpatients at a university hospital. Healthcare 13:245. doi: 10.3390/healthcare13030245, 39942434 PMC11816970

[ref8] Bhatta D. R. Cavaco L. M. Nath G. Kumar K. Gaur A. Gokhale S. . (2016). Association of Panton Valentine Leukocidin (PVL) genes with methicillin resistant *Staphylococcus aureus* (MRSA) in Western Nepal: a matter of concern for community infections (a hospital based prospective study). BMC Infect. Dis. 16:199. doi: 10.1186/s12879-016-1531-1, 27179682 PMC4867903

[ref9] Bourgeois-Nicolaos N. Rouard C. Desroches M. Doucet-Populaire F. (2012). Résistance au linézolide chez les staphylocoques. J. Anti-Infect. 14, 108–115. doi: 10.1016/j.antinf.2012.07.004

[ref10] Deplano A. Witte W. Leeuwen W. J. V. Brun Y. Struelens M. J. (2000). Clonal dissemination of epidemic methicillin-resistant *Staphylococcus aureus* in Belgium and neighboring countries. Clin. Microbiol. Infect. 6, 239–245. doi: 10.1046/j.1469-0691.2000.00064.x, 11168119

[ref11] Eckhardt C. Halvosa J. S. Ray S. M. Blumberg H. M. (2003). Transmission of methicillin-resistant *Staphylococcus aureus* in the neonatal intensive care unit from a patient with community-acquired disease. Infect. Control Hosp. Epidemiol. 24, 460–461. doi: 10.1086/502234, 12828327

[ref12] EUCAST. European Committee on Antimicrobial Susceptibility Testing (EUCAST). (2025). Available online at: https://www.eucast.org/ (Accessed 24 November, 2025).

[ref13] European Centre for Disease Prevention and Control WHO publishes list of bacteria for which new antibiotics are urgently needed. (2017). Available online at: https://www.who.int/news/item/27-02-2017-who-publishes-list-of-bacteria-for-which-new-antibiotics-are-urgently-needed. (Accessed 03 February, 2026).

[ref14] Gebremeskel F. T. Alemayehu T. Ali M. M. (2022). Methicillin-resistant *Staphylococcus aureus* antibiotic susceptibility profile and associated factors among hospitalized patients at Hawassa university comprehensive specialized hospital, Ethiopia. IJID Reg. 3, 129–134. doi: 10.1016/j.ijregi.2022.03.015, 35755464 PMC9216632

[ref15] Gillet Y. Issartel B. Vanhems P. Fournet J. C. Lina G. Bes M. . (2002). Association between *Staphylococcus aureus* strains carrying gene for Panton-Valentine leukocidin and highly lethal necrotising pneumonia in young immunocompetent patients. Lancet 359, 753–759. doi: 10.1016/S0140-6736(02)07877-7, 11888586

[ref16] Gondi Nzamba B. F. Otsaghe Ekore D. Dikoumba A. C. Nzue Nguema J. Ntsaga Matamba E. C. Dibakou S. E. . (2025). Characterization and diversity of methicillin-resistant *Staphylococcus aureus* in two hospitals in Gabon. BMC Infect. Dis. 25:1332. doi: 10.1186/s12879-025-11727-3, 41094358 PMC12522221

[ref17] Gupta N. Abd El-Gawaad N. S. Mallasiy L. O. (2024). Hospital-borne hazardous air pollutants and air cleaning strategies amid the surge of SARS-CoV-2 new variants. Heliyon 10:e38874. doi: 10.1016/j.heliyon.2024.e38874, 39449698 PMC11497388

[ref18] Harikripal Z. S. P. Sie A. Ouedraogo A. Danquah I. Bhattacharyya S. (2026). Rural–urban migration and low-transmission endemicity of malaria in sub-Saharan Africa: a statistical inference approach to uncover possible mechanisms. J. Epidemiol. Glob. Health 16:16. doi: 10.1007/s44197-025-00513-8, 41670913 PMC12921076

[ref19] Hesari M. R. Salehzadeh A. Darsanaki R. K. (2018). Prevalence and molecular typing of methicillin-resistant *Staphylococcus aureus* carrying Panton-Valentine leukocidin gene. Acta Microbiol. Immunol. Hung. 65, 93–106. doi: 10.1556/030.64.2017.032 28859499

[ref20] Kawayanagi T. Kawada-Matsuo M. Kusaka S. Yasutomi Y. Suzuki Y. Nishihama S. . (2025). Clinical and genetic analysis of oral and nasal *Staphylococcus aureus* isolates in dental patients. Sci. Rep. 15:13149. doi: 10.1038/s41598-025-93773-0, 40240397 PMC12003906

[ref21] Kpeli G. Buultjens A. H. Giulieri S. Owusu-Mireku E. Aboagye S. Y. Baines S. L. . (2017). Genomic analysis of ST88 community-acquired methicillin resistant *Staphylococcus aureus* in Ghana. PeerJ 5:e3047. doi: 10.7717/peerj.3047, 28265515 PMC5333547

[ref22] Linde H. Wagenlehner F. Strommenger B. Drubel I. Tanzer J. Reischl U. . (2005). Healthcare-associated outbreaks and community-acquired infections due to MRSA carrying the Panton-Valentine leucocidin gene in southeastern Germany. Eur. J. Clin. Microbiol. 24, 419–422. doi: 10.1007/s10096-005-1341-7, 15937659

[ref23] Lowy F. D. (1998). *Staphylococcus aureus* infections. N. Engl. J. Med. 339, 520–532. doi: 10.1056/NEJM199808203390806, 9709046

[ref24] MacGillivray D. M. Kollmann T. R. (2014). The role of environmental factors in modulating immune responses in early life. Front. Immunol. 5:434. doi: 10.3389/fimmu.2014.00434, 25309535 PMC4161944

[ref25] Magiorakos A. P. Srinivasan A. Carey R. B. Carmeli Y. Falagas M. E. Giske C. G. . (2012). Multidrug-resistant, extensively drug-resistant and pandrug-resistant bacteria: an international expert proposal for interim standard definitions for acquired resistance. Clin. Microbiol. Infect. 18, 268–281. doi: 10.1111/j.1469-0691.2011.03570.x, 21793988

[ref26] Maltezou H. C. Giamarellou H. (2006). Community-acquired methicillin-resistant *Staphylococcus aureus* infections. Int. J. Antimicrob. Agents 27, 87–96. doi: 10.1016/j.ijantimicag.2005.11.004 16423509

[ref27] Masson E. (2026). Saisonnalité des infections cutanées à *S. aureus* et ajustement de l’antibiothérapie empirique. Available online at: https://www.em-consulte.com/article/1775183/saisonnalite-des-infections-cutanees-a-s-aureus-et (Accessed 08 February, 2026).

[ref28] Ngoubangoye B. Fouchet D. Boundenga L. A. Cassan C. Arnathau C. Meugnier H. . (2023). *Staphylococcus aureus* host Spectrum correlates with methicillin resistance in a multi-species ecosystem. Microorganisms 11:396. doi: 10.3390/microorganisms11020393, 36838358 PMC9964919

[ref29] Pietrocola G. Nobile G. Rindi S. Speziale P. (2017). *Staphylococcus aureus* manipulates innate immunity through own and host-expressed proteases. Front. Cell. Infect. Microbiol. 7:166. doi: 10.3389/fcimb.2017.00166, 28529927 PMC5418230

[ref30] Schaumburg F. Köck R. Friedrich A. W. Soulanoudjingar S. Ngoa U. A. von Eiff C. . (2011). Population structure of *Staphylococcus aureus* from remote African Babongo pygmies. PLoS Negl. Trop. Dis. 5:e1150. doi: 10.1371/journal.pntd.0001150, 21572985 PMC3091839

[ref31] Shume T. Urgesa K. Mekonnen S. Ayele F. Tesfa T. Tebeje F. . (2024). Nasal carriage of MRSA among clinically affiliated undergraduate students at the College of Health and Medical Sciences, Haramaya University, Ethiopia. Sci. Rep. 14:29977. doi: 10.1038/s41598-024-80794-4, 39622867 PMC11612472

[ref32] Tadesse B. T. Ashley E. A. Ongarello S. Havumaki J. Wijegoonewardena M. González I. J. . (2017). Antimicrobial resistance in Africa: a systematic review. BMC Infect. Dis. 17:616. doi: 10.1186/s12879-017-2713-1, 28893183 PMC5594539

[ref33] Tattevin P. (2011). Community-acquired methicillin-resistant *Staphylococcus aureus* (MRSA) infections. Med. Mal. Infect. 41, 167–175. doi: 10.1016/j.medmal.2010.11.017, 21215538

[ref34] Turner N. A. Sharma-Kuinkel B. K. Maskarinec S. A. Eichenberger E. M. Shah P. P. Carugati M. . (2019). Methicillin-resistant *Staphylococcus aureus:* an overview of basic and clinical research. Nat. Rev. Microbiol. 17, 203–218. doi: 10.1038/s41579-018-0147-4, 30737488 PMC6939889

[ref35] Velasco V. Sherwood J. S. Rojas-García P. P. Logue C. M. (2014). Multiplex real-time PCR for detection of *Staphylococcus aureus*, *mecA* and Panton-Valentine Leukocidin (PVL) genes from selective enrichments from animals and retail meat. PLoS One 9:e97617. doi: 10.1371/journal.pone.0097617, 24849624 PMC4029734

[ref36] Wertheim H. F. L. Melles D. C. Vos M. C. van Leeuwen W. van Belkum A. Verbrugh H. A. . (2005). The role of nasal carriage in *Staphylococcus aureus* infections. Lancet Infect. Dis. 5, 751–762. doi: 10.1016/S1473-3099(05)70295-4, 16310147

